# Spectroscopy of carrier multiplication in nanocrystals

**DOI:** 10.1038/srep20538

**Published:** 2016-02-08

**Authors:** Benjamin Bruhn, Rens Limpens, Nguyen Xuan Chung, Peter Schall, Tom Gregorkiewicz

**Affiliations:** 1 Van der Waals-Zeeman Institute, University of Amsterdam, Science Park 904, 1098 XH Amsterdam, The Netherlands

## Abstract

Carrier multiplication in nanostructures promises great improvements in a number of widely used technologies, among others photodetectors and solar cells. The decade since its discovery was ridden with fierce discussions about its true existence, magnitude, and mechanism. Here, we introduce a novel, purely spectroscopic approach for investigation of carrier multiplication in nanocrystals. Applying this method to silicon nanocrystals in an oxide matrix, we obtain an unambiguous spectral signature of the carrier multiplication process and reveal details of its size-dependent characteristics-energy threshold and efficiency. The proposed method is generally applicable and suitable for both solid state and colloidal samples, as well as for a great variety of different materials.

Carrier multiplication (CM) occurs when multiple excitons are created upon the absorption of a single high-energy photon. This mechanism promises increases in photodetector sensitivity^1^, enhanced gain in lasers^2^ and a more efficient photocatalysis and photovoltaic conversion^3^,^4^,^5^.

While CM in bulk semiconductors takes place via impact ionization^6^ – by transferring excess energy of a high-energy hot carrier to a valence band electron^7^,^8^ – it is inherently inefficient because of momentum conservation restrictions and competition with phonon scattering. In nanostructures, by contrast, several quantum confinement-related effects increase the CM rate^9^. To date, efficient CM has been reported for nanocrystals of both direct (e.g. CdSe^10^, PbS^11^, PbSe^10^,^11^,^12^,^13^,^14^,^15^,^16^, InAs^17^,^18^ and CdTe^19^) and indirect (e.g. Si^20^ and Ge^21^) bandgap materials, in nanowires^8^, nanosheets^22^, and also for carbon nanotubes^23^ and in graphene^24^,^25^,^26^,^27^,^28^. Dedicated experiments revealed that CM proceeds on sub-picosecond time-scale^29^, in agreement with theoretical modeling^30^. Since the multiple excitons (that are created by CM) are typically short-lived due to efficient Auger recombination^30^, the existence of the CM in nanostructures is mostly investigated by ultrafast optical techniques, like transient induced absorption, terahertz and upconversion spectroscopies, and also by photocurrent measurements^4^. For dense solid-state dispersions of silicon nanocrystals in silicon dioxide, it has been shown that multiple excitons can escape nonradiative recombination by separating into proximal nanocrystals; in this case, CM can increase the quantum yield of excitonic photoluminescence^20^,^31^,^32^. However, both approaches-the ultrafast optical spectroscopy and the photoluminescence quantum yield determination-are experimentally challenging and rely on comparison of calibrated signal intensities, hence they are prone to artifacts and misinterpretations^33^. In fact, it turned out that some of the initial reports overestimated the efficiency of CM or could not be reproduced^18^,^34^,^35^,^36^,^37^,^38^. While these issues could partly be traced back to experimental deficiencies and differences in nanocrystal fabrication processes or surface chemistry^10^,^11^,^12^,^13^,^14^,^15^,^16^,^17^,^18^,^20^,^21^, evidence is still mostly comprised of the accumulating number of publications reporting on CM being greater than the number of publications denying its existence. An independent, unambiguous proof for CM would help end this ongoing discussion^33^.

In this paper we propose a new experimental approach for the investigation of CM in nanocrystals, alternative to the methods applied in the past and free from their deficiencies, and demonstrate the unique spectral signature of CM. In that way we obtain an independent evidence of CM that does not rely on intensity arguments, thus confirming its occurrence beyond reasonable doubt. Moreover, we explicitly demonstrate that the threshold energy and possibly also the efficiency of CM are functions of the bandgap, and consequently the size of nanocrystals, within a particular ensemble. These essential insights into the CM process are directly relevant to its physical mechanism and represent a unique advantage of the spectroscopic method developed in this study. The method itself is of general character and can be applied to any ensemble of nanocrystals, which is not monodisperse, and where emission due to multiple excitons is possible^39^.

## Results and Discussion

**Figure 1:**
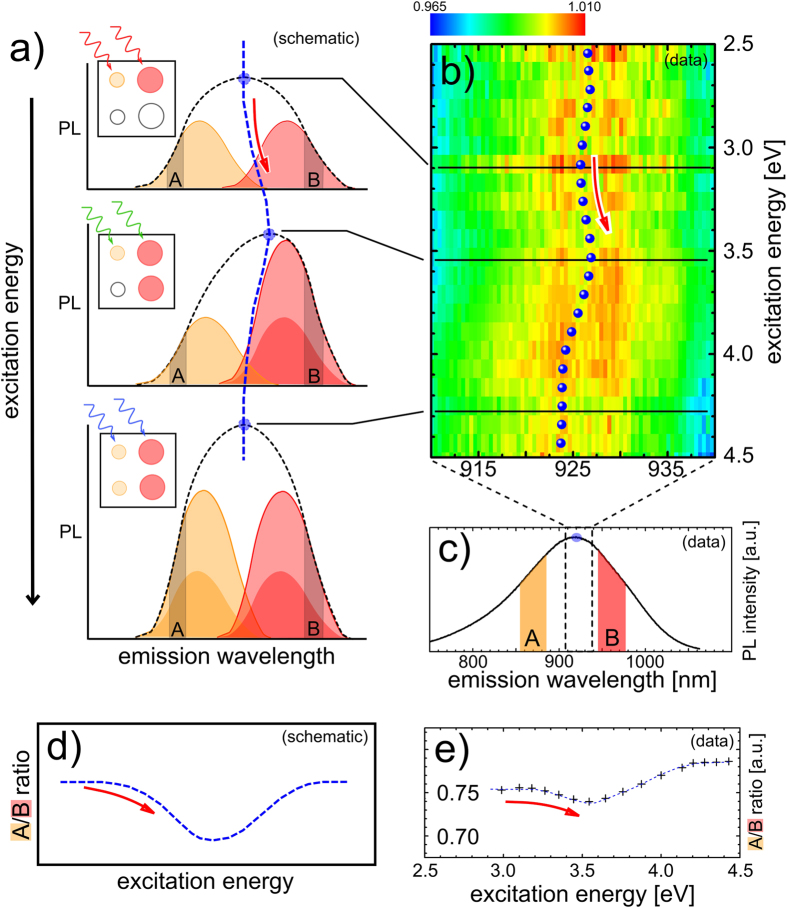
(**a**) Simplified schematic representation of a nanocrystal ensemble spectrum comprised of contributions from nanocrystals of different sizes. The shaded areas denoted as A and B can be seen as an approximate representation of such (see (**c–e**) for the use of these). As shown in the insets, in the top panel the excitation energy does not surpass any CM threshold, in the mid panel large nanocrystals exhibit CM, and in the bottom panel all nanocrystals participate in CM. The spectral shape, and therefore the peak position, changes from step to step, indicated by the blue dashed line. (**b**) Experimentally obtained spectra of a nanocrystal ensemble (sample #1) as a function of excitation energy. The three different cases sketched in (**a**) are indicated as black horizontal lines. The peak position, obtained by fitting and indicated by blue circles, exhibits a red-shift, as indicated by the red arrow, followed by a recovery. (**c**) Photoluminescence spectrum showing the whole emission range, of which only a part, marked by a dashed rectangle, was depicted in (**b**). Two colored areas indicate the parts used for the ratio analysis described in (**d**). (**d**) A schematic representation of the ratio of the emission contribution of the two nanocrystals populations, qualitatively showing a similar pattern as the peak position. (**e**) Intensity ratio of the contributions indicated by colored rectangles in (**c**). A clear change can be observed, showing the same pattern as the peak position in (**b**), similar to the schematic in (**a**).

While direct bandgap quantum dots allow for multiple-exciton generation and radiative biexciton recombination competing with non-radiative processes^40^, indirect bandgap materials like silicon, due to long exciton lifetimes, do not exhibit increased emission through multiple-exciton generation within the same nanocrystal; however, it has been shown^32^ that sufficiently high excess energy in one nanocrystal can undergo an ultrafast transfer to a neighboring nanocrystal, where another exciton is created. This happens on a time scale shorter than or comparable to energy loss mechanisms. Consequently, CM is reflected in photoluminescence quantum yield here as well. The samples used in this study – dense solid-state dispersions of silicon nanocrystals in silicon dioxide – are similar to samples in previous work^20^,^31^,^32^, for which CM has been demonstrated using other techniques. The new approach proposed here builds upon a presumption that, since CM involves exciton generation, its energy threshold should be correlated to the bandgap value, and therefore to the nanocrystal size. For large nanocrystals, CM should commence at a lower excitation energy than for the smaller ones, due to correspondingly higher excess energy. Therefore, the photoluminescence spectrum of an ensemble of nanocrystals is expected to change as the CM sets in, due to the enhanced contribution of larger grains. In that way a purely “spectral” signature of CM could appear, where evidence of the CM process manifests itself as a change of the photoluminescence spectrum rather than an experimentally challenging quantum yield enhancement or a modification of a rather unspecific utrafast absorption transient. This idea is schematically illustrated in Fig. 1a: as the excitation energy increases above the threshold of CM, an enhancement of photoluminescence appears-first from larger nanocrystals (at a longer emission wavelength), followed by the contribution from the smaller ones. Effectively, the initial onset of CM from the large nanocrystals induces an apparent red-shift of the spectrum, which then gradually disappears as larger and larger parts of the nanocrystal ensemble contribute to the CM process at higher excitation energies. This is indicated by the dashed line that traces the maximum of the spectrum; its red-shift mirrors the modification of the entire spectrum. It should be noted here that spectral shifts are only an indirect measure of the processes at work, and that the enhancement of a part of the spectrum is the primary effect. Thus, only quantification of the latter really has a meaning in this context.

Experimental data, displayed in Fig. 1b, indeed confirm the expectation just explained. The spectra, plotted as a function of excitation energy, with color indicating the intensity at the corresponding emission wavelength, reveal a distinct red-shift, as indicated in Fig. 1a. We highlight this shift by using blue dots to trace the interpolated peak maximum. Indeed, both the underlying spectrum and the extrapolated maximum show a small (typically in the order of a few nanomerters), but clearly distinguishable shift, in agreement with the sketch in Fig. 1a.

In experimental practice, and for the sake of using a direct measure of the investigated phenomenon, this spectral shift can be conveniently and accurately monitored by plotting the ratio of photoluminescence from two fractions of the nanocrystal ensemble – large and small ones – allowing for sensitive probing of differences in their (relative) contributions to the total emission. This is illustrated in Fig. 1c, where we highlight two parts of the spectrum, one on each side of the maximum, and Fig. 1d, where a schematic representation of the corresponding ratio is shown. The spectrum shifts as the contributions of the two parts change, leading to a shift of their area ratio. Indeed, plotting this area ratio as a function of excitation energy (Fig. 1e) reveals a clear spectral shift, in agreement with Fig. 1b. In this way, the effect can be measured precisely, as two parts of the same photoluminescence spectrum are compared, obliterating the need for any intensity calibration. In fact, the experimental error is well below the size of the data symbols in Fig. 1e. Note that the experiment is conducted under low excitation photon flux (with the average number *N* of photons absorbed per nanocrystal being much less than unity, typically *N* < 0.01 for the results presented here), in order to avoid any photoluminescence saturation^20^ effects. Also, in the investigated material the spectral shift is accompanied by the increase of photoluminescence quantum yield as expected for CM, which excludes its possible interpretation in terms of absorption peculiarities or saturation effects-see the Supplementary Information for further details. The method can even be used on samples that suffer from decreasing ensemble quantum yield as a function of excitation energy (which is commonly observed in colloidal nanocrystal samples due to, e.g., hot carrier trapping).

**Figure 2:**
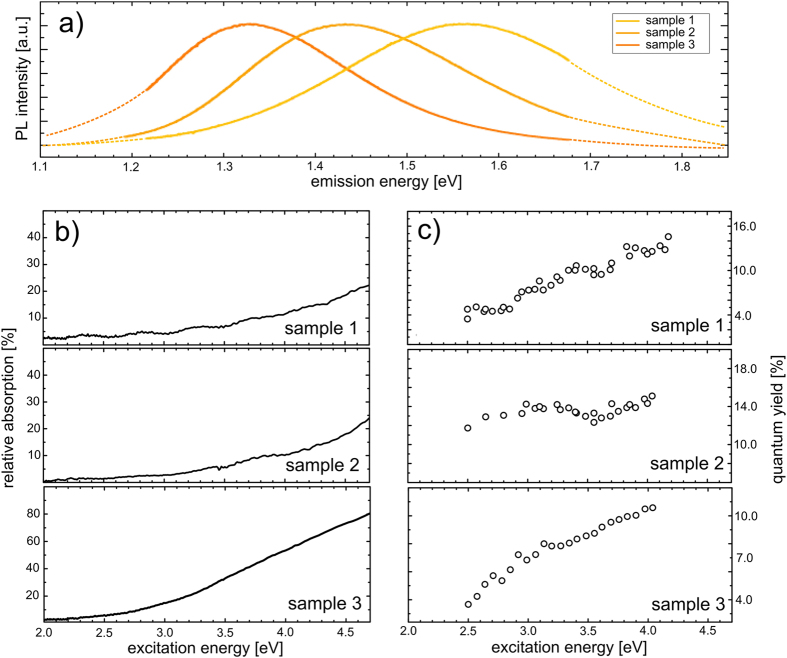
(**a**) Photoluminescence spectra of the three samples used in this work. (**b**) Absorption spectra. (**c**) Quantum yield as a function of excitation wavelength.

To reveal the potential of the CM spectroscopy developed in this study, we choose three samples that differ in average nanocrystal size, as reflected in their photoluminescence spectra (see Fig. 2). All of them are multinanolayer structures known to feature superior optical properties and a narrow nanocrystal size distribution^41^,^42^; they are comprised of 100 layers of two-dimensional sheets of silicon nanocrystals, separated by 5 nm spacers of pure silicon dioxide. The nanocrystals have average sizes of 2.8, 3.5 and 4.8 nanometers for samples #1 to #3, respectively, as deduced from the photoluminescence spectra based on the structural analysis of Takeoka *et al.*^43^. A clear increase of the quantum yield with excitation energies is observed for all the investigated samples (see Fig. 2).

**Figure 3:**
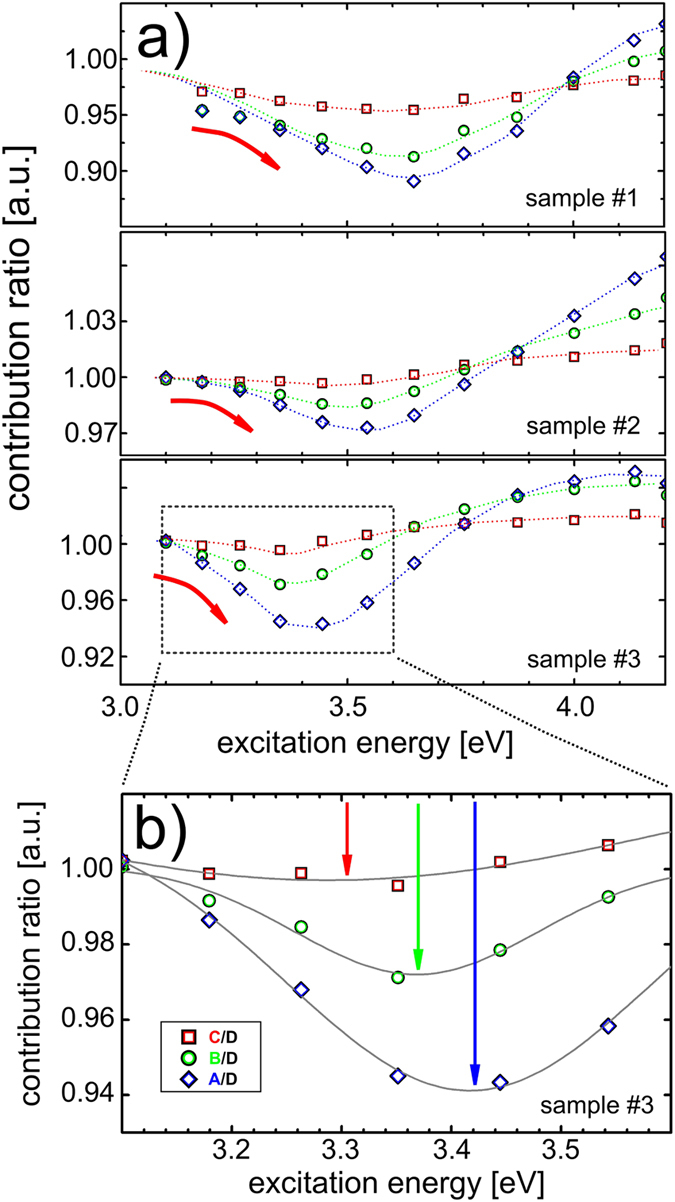
(**a**) Intensity ratios of different nanocrystal populations A (highest emission energy) to D (lowest emission energy), see legend in (**b**) for color codes and inset in Fig. 4 for a schematic representation. The CM spectral signature shown in Fig. 1 can be observed for all samples. (**b**) Magnification of the part marked by a dashed rectangle in (**a**). Peak position (indicated by arrows) and depth clearly shift, following the spectral signature expected from CM. See Fig. 4 for the corresponding modelling. (**a,b**) The errors are within the data symbols.

We show the evolution of photoluminescence spectra with excitation energy, as measured by the area ratio of four photon energy ranges (see Supplementary Information for actual details), in Fig. 3. Clearly, the results for all samples feature the initial red-shift of the ensemble photoluminescence spectrum with excitation energy, in line with the expectation sketched in Fig. 1 for the CM process. As before, the ratios are plotted in such a way that a decrease reflects the enhanced contribution from larger nanocrystals, and therefore the “red”-shift of the total photoluminescence spectrum to lower energies. We note that the shift and its magnitude are enhanced for photoluminescence contributions that are further apart, reflecting a consistent modification of the entire spectrum. The fine structure of these shifts, as evident in the enlarged section shown in Fig. 3b, differs between all samples, evidencing intimate differences of the CM process in a particular ensemble.

**Figure 4:**
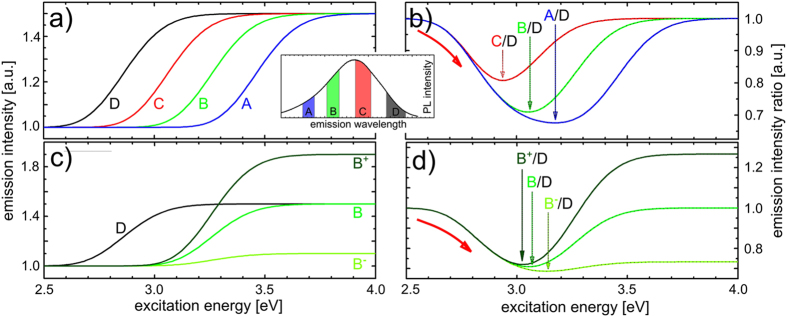
(**a**) Emission intensity of the nanocrystal populations whose photoluminescence contributions are indicated in the inset, normalized to their respective emission intensity below the CM threshold. 50% CM efficiency is being assumed for all NC sizes. (**b**) Emission intensity ratios calculated from the curves in (**a**) The temporary decrease (negative peak) shifts in position and magnitude when the difference between the compared fractions is larger. (**c**) Emission intensity of nanocrystal populations B and D (see inset), where the emission of B increases less than (B−), equally to (B), and more than (B+) that of D. (**d**) Emission intensity ratios calculated from the curves in (**c**). The ratio exhibits distinctly different behavior, depending on the difference in CM efficiency of the individual populations.

In order to fully explore and appreciate the power of the spectroscopic approach developed in this study, we construct a simple model of the spectral shift. We focus on four nanocrystal populations of an ensemble corresponding to the spectral contributions schematically depicted in Fig. 4a (inset), and model their emission intensities separately as a function of excitation energy. Note that a fit of the spectrum of sample #3 is used for the model calculations. Figure 4a shows the emission of the different nanocrystal populations as a function of excitation energy, normalized to the contribution at low excitation energy. For the larger nanocrystals, CM sets in earlier, which is reflected in the earlier rise of the emission intensity. The resulting emission intensity ratio displayed in Fig. 4b indeed shows a characteristic red-shift, in close agreement with the experimental measurements. It also readily reproduces the systematic shift of the minimum to higher excitation energies for more separate nanocrystal sizes as shown in Fig. 3b. In our model, this results from the longer build-up of the increasing contribution of big nanocrystals before the enhanced emission from smaller ones sets in. Thus, the data in Fig. 3b provide direct experimental evidence that the CM threshold for nanocrystals within a particular ensemble is correlated with their bandgap (e.g size)-something which could not be explicitly shown in previous studies, using the “traditional” approach^32^. Please note that an excess energy of approximately 250 meV needs to be added, which may be interpreted as a Stokes-shift, in order to reach an agreement between the modelling and the experimental data. Moreover, this CM threshold may be a function of nanocrystal size, or emission energy, which this new analysis method allows to investigate and account for. Additionally, the preliminary analysis of the data in Fig. 3 indicates that the actual excess energy (above multiples of the bandgap energy) decreases for larger bandgaps. Therefore, energy-wise the CM process becomes more efficient for smaller nanocrystals. This is an important conclusion uniquely revealed by the spectroscopic approach.

To understand further differences between CM in the three investigated samples, Fig. 3, and specifically the different magnitude of the subsequent “blue” shift marking completion of the CM process, we also model the effect of size-dependent efficiency of the multiplication process (which has e.g. been observed in PbS and PbSe nanocrystals^44^), being lower, equal or higher for the small nanocrystals with respect to the largest ones, as shown in Fig. 4c. Clearly, this leads to different final values of the emission intensity ratios, as well as a systematic shift of the minimum value of the measured ratio, see Fig. 4d. Taking both effects together, we can now rationalize the full behavior of the spectra in Fig. 3. The ratios of different contributions to the photoluminescence spectrum indeed show a shift of their minimum in excitation energy (with respect to each other), as well as a difference in their final values at high excitation energy. In that way, the size distribution of nanocrystals together with their size-dependent CM efficiency within the ensemble are directly visualized, and can be experimentally investigated. We note that this detailed characterization of CM was not available with the previously used experimental approaches, and represents a unique advantage of the novel method developed here.

## Conclusions

Making use of a novel, purely spectroscopic approach, we provide independent and conclusive evidence of CM in semiconductor nanocrystals and demonstrate its size-dependent characteristics. The proposed method is highly reliable and free of experimental hurdles related to those applied in the past, not requiring tedious determination of the absolute quantum yield of photoluminescence and/or calibrated ultrafast transient optical spectroscopy in weakly absorbing media. It is not material-specific and can be used to investigate CM in ensembles of any centers which support emission of multiple excitons – either directly or by excitation transfer. We demonstrate the power of this approach by attesting CM in layers of closely packed silicon nanocrystals embedded in a silicon dioxide matrix. In this case we explicitly demonstrate the nanocrystal size dependence of the energy threshold and the efficiency of the CM process-an information highly relevant for theoretical modeling of this important phenomenon. In more general terms, the developed strategy can be expanded for detailed characterization of complex ensemble spectra of various origins.

## Methods

The experiments have been conducted on silicon nanocrystal multinanolayer structures. The nanocrystals were dispersed in a silicon dioxide matrix by magnetron co-sputtering using high purity Si (99.99%) and SiO_2_ (99.99%) targets. The sputtered films (stacks of 3.5 nm thick active SiO_*x*_ and 5 nm thick passive SiO_2_ layers, 500 nm thick in total) were annealed for 0.5 h at temperatures ranging from 1250 to 1150 °C in nitrogen atmosphere and have excess Si amounts from 30–12 Vol%. The high temperature heat treatment induces phase separation between Si and SiO_2_, forming Si nanocrystals. By investigating set of samples – #1 (1250 °C and 30% excess Si), #2 (1200 °C and 25% excess Si) and #3 (1150 °C and 12% excess Si) – a broad range of nanocrystal sizes was investigated.

Photoluminescence measurements were performed using a xenon lamp, a Solar LS M130 monochromator and an optical band pass filter (to avoid second order excitation light being detected as emission from the sample) for excitation, and an optical long pass filter (to remove the excitation source), a Solar LS M266 spectrometer and a Hamamatsu S7031-1008S CCD camera for detection. The measured spectra were corrected for the system response.

## Additional Information

**How to cite this article**: Bruhn, B. *et al.* Spectroscopy of carrier multiplication in nanocrystals. *Sci. Rep.* **6**, 20538; doi: 10.1038/srep20538 (2016).

## Supplementary Material

Supplementary Information
