# Supplementary material for: Spectroscopy of carrier multiplication in nanocrystals
Source: Sci Rep. 2016 Feb 8;6:20538. doi: 10.1038/srep20538 (PMC4744935; doi:10.1038/srep20538)
Supplement: Supplementary Information [file srep20538-s1.pdf]

Supplementary Information

This is the Supplementary Information section for the article *Spectroscopic signature of carrier multiplication in nanocrystals* by the authors *Bruhn, Limpens, Chung, Schall and Gregorkiewicz.*

Additional Experimental Methods

External quantum yield (EQY) measurements were performed in an integrating sphere in order to avoid possible influences of light scattering and reflections, as well as inhomogeneous spatial distribution of emission. A xenon lamp combined with a Solar LS M130 monochromator and an optical band pass filter were used for excitation, while the emission was detected using an optical long pass filter, a Solar LS M266 spectrometer and a Hamamatsu S7031-1008S CCD camera. All spectra were corrected for the system response. Absorption measurements were carried out using a Perkin Elmer UV-VIS Lambda900 spectrometer in combination with an integrating sphere, accounting for reflection and scattering effects.

Populations for ratio analysis

For the ratio analysis described in the manuscript four nanocrystal populations with photoluminescence contributions A-D have to be selected. The integral intensity of each interval is extracted and used to calculate the ratios. Fig. S1 shows which intervals were chosen for each sample. The corresponding results are displayed in

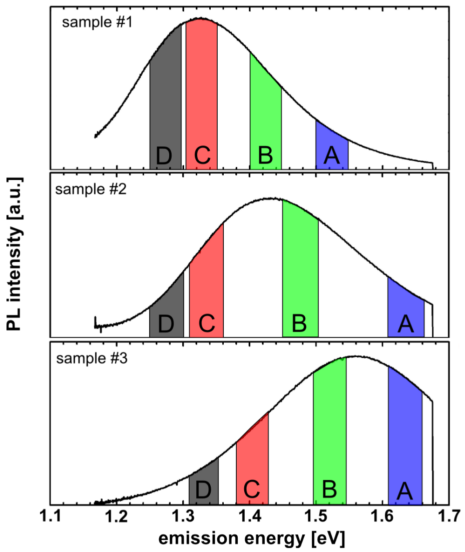

Figure S1: Spectra and respective energy ranges chosen for the ratio analysis.

Fig.2 of the manuscript.

The energy ranges for the extraction of emission intensities have been chosen individually for each sample, so that D is at the lowest possible emission energy, but still maintains a reasonable S/N ratio, C is close to D, B is roughly equidistant to C and A, and A is at the highest possible emission energy, while maintaining a reasonably good S/N ratio.

Data analysis

While the peak position shift is the most obvious indicator of spectral changes, its analysis cannot yield very detailed information about the processes that cause it. In fact, it is rather an indirect measure of the spectral changes that occur with increasing excitation energy, caused by changing contributions to the PL of different NC size populations within the ensemble. Therefore, the contributions shall be used for analysis directly.

For a PL spectrum at a specific excitation energy, the spectral range of the emission window is split into

intervals of equal energy. The spectrally resolved emission intensity is integrated within each interval, through which the PL contribution of different NC-size populations is obtained. Subsequently, the ratio of the PL intensities of two specific intervals is calculated (as the contribution of smaller NCs divided by the contribution of larger NCs), representing the relative emission intensity change of two sub-populations of the ensemble. In that way, normalization to the excitation intensity is carried out automatically.

These steps are performed for a number of excitation energies. The ratios can then be plotted as a function of excitation energy. A constant means that the contributions do not change relative to each other, a decrease means that the contribution of larger NCs increases (also seen as a red-shift of the peak maximum), and an increase is caused by an increasing contribution of smaller NCs (naturally leading to a blue-shift of the maximum of the PL spectrum).

The error in the determination of the ratio value is defined by the signal-to-noise ratio of the spectrum in the corresponding range of the two extraction regions. It is typically very small and results in a more accurate description than the peak position change, especially since the latter depends on fitting of the photoluminescence spectrum, which cannot necessarily be described well by a simple mathematical function like e.g. a normal distribution.

Discussion of Spectral and Quantum Yield Changes

In this section, we will highlight the factors that can have an influence on the spectral shape of NC ensemble PL and external quantum efficiency and will rule out explanations alternative to carrier multiplication for the unique spectral signature presented in this work.

As discussed earlier, by using two regions from the same spectrum, the excitation intensity influence is automatically removed. Given an ensemble with a fixed size distribution, that leaves three factors that can alter the shape of the ensemble PL spectrum and the external quantum yield with increasing excitation energy:

First of all, the size-dependent absorption cross section (ACS) defines which fraction of the light is absorbed by different NC-size populations. If the ACS of one population as a function of excitation energy increases faster than that of another, their relative contributions to the ensemble PL spectrum change. It is, however, well-established that the ACS of different Si NCs increases at the same rate at high excitation energies, which removes the influence of ACSs from the ratio curve. Moreover, even if there was a size-dependent absorption edge, related, for example, to the direct bandgap transition in Si NCs, it would occur first for smaller NCs [1], leading to an increasing contribution of small NCs at lower excitation energies than for large NCs. Additionally, if the spectral signature described in this work would originate from increasing absorption of large NCs (relative to smaller NCs), then a decrease of QY would appear, which is not observed. It has been shown in the past, that EQY of Si NCs increases with decreasing size, until reaching a maximum and then collapsing for ultrasmall NCs due to their inferior quality [2] [3]. Typical EQY values (for co-sputtered Si NCs samples) can be found in references [4–6]. Samples used in this work typically do not contain significant amounts of very small NCs, therefore (in our case) smaller NCs generally exhibit higher average quantum yield than the larger ones. Therefore, an increasing absorption of large NCs relative to smaller NCs naturally leads to a decrease in the ensemble QY, which is experimentally not observed.

Another factor that could cause a spectral change, which has been precluded by carefully designing the experiment at low excitation intensity, but should nevertheless be kept in mind, is PL saturation. Large NCs have a higher ACS than small ones, so that they saturate first. Therefore, saturation would lead to an increasing contribution of small NCs, while at the same time decreasing the ensemble QY. In the current study, the experiments have been performed under very low excitation, with the average number of photons absorbed per NC being $N_x \gg 0.01$, i.e. very far from the excitation regime where saturation (through 2-photon absorption) could commence.

To summarize this section, since the quantum yield of small NCs is larger than that for large ones, as proven

in the past [5,6], only carrier multiplication (with a size-dependent threshold, which is lower for large NCs) can explain the appearence of the red shift and the spectral feature considered in this study.

References

[1] W.D.A.M. de Boer, D. Timmerman, K. Dohnalova, I.N. Yassievich, H. Zhang, W.J. Buma, and T. Gregorkiewicz. Red spectral shift and enhanced quantum efficiency in phonon-free photoluminescence from silicon nanocrystals. *Nature Nanotechnology*, 5:878–884, 2010.

[2] R. Limpens and T. Gregorkiewicz. Spectroscopic investigations of dark Si nanocrystals in SiO_2 and their role in external quantum efficiency quenching. *Journal of Applied Physics*, 114(7):074304, 2013.

[3] K. Dohnalova, T. Gregorkiewicz, and K. Kusova. Silicon quantum dots: surface matters. *Journal of Physics: Condensed Matter*, 26(17):173201, 2014.

[4] R. Limpens, A. Lesage, M. Fuji, and T. Gregorkiewicz. Size confinement of si nanocrystals in multinanolayer structures. *Scientific Reports*, 5:17289, 2015.

[5] H. Sugimoto, M. Fujii, K. Imakita, S. Hayashi, and K. Akamatsu. Codoping n-and p-type impurities in colloidal silicon nanocrystals: Controlling luminescence energy from below bulk band gap to visible range. *Journal of Physical Chemistry C*, 117(22):11850–11857, 2013.

[6] W. Sun, C. Qian, L. Wang, M. Wei, M.L. Mastronardi, G. Casillas, J. Breu, and G.A. Ozin. Switching-on quantum size effects in silicon nanocrystals. *Advanced Materials*, 2014.
